# A Theoretical Development of the Gender Embodiment of Enrichment: A Study of Gender Norms in Enrichment and Factors Related to Enrichment in a Sample of the Swedish Working Population

**DOI:** 10.3389/fsoc.2021.669789

**Published:** 2021-04-29

**Authors:** Emma Hagqvist, Anna Nyberg, Constanze Leineweber

**Affiliations:** ^1^Department of Psychology, Stress Research Institute, Stockholm University, Stockholm, Sweden; ^2^Department of Public Health and Caring Sciences, Uppsala University, Uppsala, Sweden; ^3^Unit of Occupational Medicine, Institute of Environmental Medicine, Karolinska Institutet, Stockholm, Sweden

**Keywords:** enrichment, factor structure, gender, theoretical development, working population, Sweden

## Abstract

Enrichment is a phenomenon described as the synergistic and beneficial effects of participating in both work and private life. Far too few studies have acknowledged the role of gender in enrichment. By applying a gender theoretical approach, this article has two aims; first, we aim to study the role of gender in enrichment by examining the factorial structure of enrichment in men and women; secondly, we aim to study the relationship between enrichment and work and private life factors in an approximately representative sample of the Swedish working population. A multigroup confirmatory factor analysis with measurement in variance was performed and this resulted in a two-factor solution for enrichment for both men and women, representing the two directions of enrichment: work-to-life enrichment (WLE) and life-to-work enrichment (LWE). Factor loadings differ across genders, indicating that men and women construct and value items of enrichment differently. Next, linear mixed models were used to answer the second aim. Results show that gendered cultural norms in work and private life manifest in the relationship between factors in the work and home sphere and enrichment. Factors in work and private life with more or less masculine or feminine epithets relate differently to WLE and LWE for men and women. The main conclusion is that masculine and feminine norms are embodied in the values and experiences of enrichment and factors related to enrichment.

## Introduction

While in the past most of the literature concerning the work–life interface has concentrated on the negative effects of having multiple roles (i.e., work-life interference), in recent years, an increasing number of studies have focused on the positive effects. The concept of work–life (or work–family) enrichment has grown as a perspective that focuses on the synergistic and beneficial effects of participating in both work and private life (Greenhaus and Powell, 2006; Allen and Martin, 2017). Enrichment is recognized as the positive side of work–life interference (Greenhaus and Beutell, 1985; Greenhaus and Powell, 2006). It is empirically suggested that enrichment works in two directions: work-to-life enrichment (WLE), and life-to -work enrichment (LWE) (Greenhaus and Powell, 2006), the two areas which encompass and, in many ways, signify gendered constructs in society. Other concepts have also been developed theoretically to express the positive linkages between private life and work exemplified by positive spill-over, enhancement, and facilitation (Carlson et al., 2006; Williams et al., 2016). In contrast to interference, where life roles have a negative impact on each other, enrichment can be explained as the extent to which one life role can improve the quality and performance in other life roles. The concept of enrichment builds on a model of a beneficial role of salience (Greenhaus and Powell, 2006; Williams et al., 2016), which argues that multiple roles (e.g., worker, spouse, and parent) are beneficial for both women and men. Likewise, it has been proposed that multiple roles are more constructive for women than for men (Cinamon and Rich, 2002). This statement is based on the general assumption that men and women have different qualities and attributes, creating a spatial and functional separation between women and men. Thus, the statement is based on gendered assumptions.

Using a constructivist theory, gender refers to the social constructions of femininities and masculinities which are expressed in what and how we behave as well as in our appearance (Connell, 2002). Gender operates through the interplay of formal and informal norms with gendered implications, which manifest in different phenomena. Williams et al. (2016) raised the concern that gender should have a more prominent place in work-life research. They argued that “[b]y neglecting gender, family-to-work enrichment measures inadvertently embrace, as uncontroversial and untroubling, some key aspects of male privilege within families.” Thus, to understand the existence and distribution of enrichment, general patterns of power, resources, inequalities, and structural conditions in work and private life must be put in focus. As we will outline in this article, the social construct of gender has an important role both in the enrichment as well as in the altered gender perspectives of factors that relate to enrichment. This renders a complex analytical model where gender cultural norms form a framework in which we view both exposure and outcome as well as the relationship between the two. The overall aim of this study is as follows: (i) to explore how gender has a key role in the construct of enrichment, and (ii) to examine the relationship between enrichment and factors in the domains of work and private life that have a strong gender cultural norm.

This study took place in Sweden. Sweden carries a gender equal norm that builds on the participation of both men and women in the labor market and their contribution to the Swedish welfare system (Hagqvist, 2016; Carbin et al., 2017). In those measures, Sweden is relatively gender equal, with a high proportion of women who participate in the labor market; 80% women compared to 85% men participate in the labor market (Statistics Sweden, 2020). Also, a relatively high number of hours in paid work by Swedish women compared to other countries (Hagqvist et al., 2017). Meanwhile, in countries with a high proportion of women in the labor market, the labor market is still highly gender-segregated (Ellingsæter, 2014) and as outlined more below, the labor market in Sweden is not omitted (Statistics Sweden, 2020).

## Theory and Concepts

### Gender and Enrichment

Social constructs and cultural expectations of gender are interwoven and penetrate into the values, appearance, culture, and symbolism of men and women, human relations, emotions, and behaviors as well as in the cultural construct of work and private life (Connell, 2002, 2006; Williams et al., 2016). Ample literature suggests that gender norms have implications on the experiences of the work-life interference of men and women (Cinamon and Rich, 2002; McGinnity and Calvert, 2009; Hagqvist et al., 2012; Fahlén, 2014). Some evidence points to a similar situation for enrichment, but here knowledge is still scarce and more research is needed (Härenstam, 2009; Williams et al., 2016; Beham et al., 2020). One of the few studies on enrichment comes from Lapierre et al. (2018), who presented marginal support for a moderating effect of gender in the relationship between enrichment and its antecedents. However, merely comparing the levels of enrichment across genders or adding gender as a moderator as pointed out by Lapierre et al. (2018) does not take gendered cultural norms into consideration. Instead, it is reasonable to assume that due to the social construction of femininities and masculinities, men and women can construct and value enrichment differently. In accordance with the aim of this study, we initially address the role of gender in enrichment and expand on the relevant theory by studying how men and women construct enrichment by testing the factorial loading of enrichment across the genders.

### Gender Cultural Norms in Factors Related to Enrichment

In accordance with the second part of the aim, we study the relationship between enrichment and factors in work and private life that are presented as gendered constructs. Specifically, in the work domain, we will focus on paid work time, management positions, industry, and employee responsibilities, and in the private life domain, we examine living with a partner, having children, time spent on housework, and perceived socioeconomic status. In the section below, we discuss the theoretical foundation to our assumptions about how these factors are gendered and how they might relate to enrichment in different ways for men and women.

Lapierre et al. (2018) concluded in their meta-analysis that factors related to enrichment are not exclusive, but tend to be domain-specific, meaning that the contexts and characteristics of the work domain tend to be more highly related to WLE than to LWE. In turn, characteristics of the family domain tend to dominantly contribute to LWE. It is important to note that Lapierre et al. (2018) had no notion of gendered cultural norms in enrichment and factors related to enrichment. Neglecting gender in factors related to enrichment could be a pitfall, abating how femininities and masculinities are expressed, reinforced, and reproduced in work and private life (West and Zimmerman, 1987). The gendered cultural norms of the work domain are embedded in everything, from the gender-specific divisions of paid and unpaid work, to how the labor market is organized and to organizational practices and policies (West and Zimmerman, 1987; Härenstam, 2009). While the work domain contests on masculine norms, the domestic and care domains contest on feminine norms. More often, women think of housework and care of others as nurturance and loving rather than work (Connell, 2002). For many women, care is a duty that they are trained for and to resist the caring of others would be an act of risking their feminine character (Huppatz, 2009). Likewise, for men, being engaged in care work can risk their manhood. Following the theory of West and Zimmerman (1987), men and women produce and reproduce gender in work (both paid and unpaid work) by enduring in masculine and feminine work tasks. As such, the masculine cultural norms in the work domain and the feminine cultural norms in the domestic and care domain are constantly reproduced. Domain specificity might therefore be not congruent for men and women.

Starting with the work domain, the norm of the good worker builds on masculinities (Hirdman, 1990; Connell, 2002; Hagqvist, 2016; Williams et al., 2016). In Sweden, a good worker is also the one who contributes to society by paying taxes (Carbin et al., 2017). Swedish studies confirm that although the difference in time spent on paid work by men and women is narrowing, men still spend marginally more time on paid work and women spend more on unpaid domestic work (Hagqvist et al., 2017, 2019; Statistics Sweden, 2020). Since paid time and un-paid time have different gendered meanings and take different numbers of hours in aspect, its relation to enrichment can vary for men and women.

According to the theories presented by Joan Acker, organizations themselves can promote masculine culture at work (Acker, 1990, 1992) creating and reinforcing both vertical and horizontal structural inequalities and disparities. These can have implications on the types of work contracts for men and women, their presence in the management positions, positions with supervisory responsibilities, and industry affinity. Terms such as “glass ceiling,” “barriers,” and “glass wall” are often used to mark the masculine culture in paid work and the structural, vertical, and horizontal inequalities that follow (Ely and Meyerson, 2000; Connell, 2006). Starting with horizontal inequalities, in Sweden, women make up more than 75% of the workforce in the public sector, while more men are found in the private sector (Martin, 2011; Statistics Sweden, 2020). Traditionally, the public sector is responsible for the care and educational work; thus feminine caring norms are also found in industry segregation. Female dominated workplaces are generally characterized by higher demands, lower decision authority (Cerdas et al., 2019), and lower control over working time (Albrecht et al., 2016). Because of this gendered horizontal division of the workforce, the employment sector could be important for the level of enrichment for men and women.

Sweden is not only horizontally segregated with male and female dominated occupations and sectors, but also vertically segregated with more men in the top posts and with high status jobs (Ely and Meyerson, 2000; Martin, 2011; Ellingsæter, 2014). Women's unequal access to the top in the Swedish labor market is visualized by the fact that men in management positions are far more numerous than women, particularly in the private sector (Statistics Sweden, 2020). Male managers in total reach 60% and in the private sector, 67% of managers are men (Statistics Sweden, 2020). The narrations concerning good management and leadership circulate around gender-appropriate behaviors building on masculine norms (Ely and Meyerson, 2000; Fogelberg Eriksson, 2016). Male and female managers are thus judged based on their gender-appropriate behaviors, while they are measured against masculine cultural norms in the management. Women who distinguish themselves from feminine-appropriate behaviors and adapt to the masculine culture in management or in paid work in general, risk losing their obligatory attributes of femininity (Huppatz, 2009). This attempt to alienate from feminine-appropriate behaviors and to adapt to the masculine culture has been seen among workers with career ambitions as well as leaders and managers (Kvande and Rasmussen, 1994; Hagqvist et al., 2020). This seems, in turn, to have had implications for how enrichment is constructed and experienced by male and female managers (Hagqvist et al., 2020). Management positions are therefore important to include in this study.

Furthermore, male and female managers are not managing on equal terms (Nyberg et al., 2015), which can also have implications for the level of enrichment. First, women are more often described as less committed to work and might not be given the same possibilities to progress to a management position as men (Ely and Meyerson, 2000). In reality, these same women are often less flexible with regard to working for long hours the companies require because they are obliged to take care of their home and children (Ely and Meyerson, 2000). Secondly, women in management positions report higher overall demands, lower influence at work, and poorer well-being than men in similar positions (Nyberg et al., 2015). Furthermore, male and female managers in the public sector tend to be clustered in different branches or industries, where the number of subordinates a manager is responsible for differs to a larger extent (Björk and Härenstam, 2016). Female managers in female industries more often have large groups of employees and less power and management support to lead these employees. To explore this further, we will study male and female managers in male- and female-dominated industries.

Moving to the private life domain, a meta-analysis indicates that family support is important for the level of experienced enrichment (Lapierre et al., 2018). Individuals who are married or cohabiting tend to report higher levels of enrichment (Lapierre et al., 2018), indicating that a partner at home can be a potential non-work support. Meanwhile, having a partner, as well as parenthood, are central aspects of life that can change gender relations. Living in a relationship means partners have to balance a powerful relationship and have constant negotiations, which tend to be based on gender role stereotypes. An emulating factor in a partnership that can contribute to a reduced role salience can among other things be an unequal division of domestic responsibilities and work. Studies show that relationships that lack equal sharing of household duties more often dissolve compared to those who share household duties (Ruppanner et al., 2018). An equal division of work tends to be more important for the family satisfaction of women than that of men (Staland-Nyman et al., 2008; Harryson et al., 2012; Eek and Axmon, 2014), and inequality tends to create deeper feelings of resentment among women than men (Bernhardt et al., 2008). Although in Sweden, there are examples of “modern couples” who aim to share work and care more equally, the societal and cultural pressure of different expectations of women and men severely hampers such ambitions among heterosexual couples (Harryson et al., 2016). While having a partner was previously proved to contribute to enrichment (Lapierre et al., 2018), exploring the relationship between having a partner and enrichment using a gender perspective can give further insights to the phenomenon.

Parenthood can strengthen gendered values between heterosexual parents, especially for women (Bernhardt et al., 2008). The norms of good motherhood are essential for femininities (DeVault, 1991; Arendell, 2000). In Sweden, parental norms imply that fathers and mothers possess the same qualifications to care for the children and the household as well as to provider activities (Elvin-Nowak and Thomsson, 2001); however, these norms seem truer for provider activities than for housework and child care (Hagqvist et al., 2017; Kling et al., 2017). Gendered parental norms are also visualized in phenomena like the weak acceptance in the Swedish labor market of fathers staying at home with children (Haas et al., 2002), the fact that the time spent by men on paid work remains unchanged when they become a parent, whereas the time spent by women reduces (Dribe and Stanfors, 2009), and the income penalization that exists of mothers but not fathers (Cantalini et al., 2017).

Gender is related to social position, another factor that could influence enrichment. Furthermore, the social position could have greater importance and significance for men than women as it provides status and power which is important in masculine hegemonies. Masculine hegemonies are hierarchies and power structures within the group of men (Connell and Messerschmidt, 2005), sometimes based on class or socioeconomic status (West and Fenstermaker, 1995; Acker, 2006). The socioeconomic position gives people societal privileges that can also have implications for the level of enrichment (Williams et al., 2016). When ranking their socioeconomic position, men seem to consider their personal income as an important factor, while women put more weight on the household financial situation (Miyakawa et al., 2012). High income gives individuals better possibilities in private life that can enrich life in general (Greenhaus and Powell, 2006). Income differences between Swedish men and women start in early life and accumulate over the lifetime (Cantalini et al., 2017) and socioeconomic status and its relationship to enrichment is therefore important to study.

## Methods

The study is based on the Swedish Longitudinal Occupational Survey of Health (SLOSH), which is a biennial self-administered questionnaire study consisting of an approximate representative sample of the Swedish working population. For details on the SLOSH population, see Magnusson Hanson et al. (2018). For the present study, data collected in 2018 were used, resulting in a total sample of 11,553 men and women who reported that they were currently in paid work. Of those, 11,468 answered all of the enrichment questions. The sample is presented in Supplementary Table 1.

Ethical approval for SLOSH was obtained from the Regional Research Ethics Board in Stockholm (Ref: 2012/373-31/5) and for the present study, from the Swedish Ethical Review Authority (Ref: 2019-00972).

### Measurements

*Enrichment* was measured using six items adapted from Fisher et al. (2009) asking whether the respondent felt that work had a positive influence on private life and whether private life had a positive influence on work. The questions are as follows:

E1 My job gives me energy to pursue activities outside of work that are important to me.
E2 Because of my job, I am in a better mood at home.
E3 The things I do at work help me deal with personal and practical issues at home.
E4 I am in a better mood at work because of everything I have going for me in my personal life.
E5 My personal life gives me the energy to do my job.
E6 My personal life helps me relax and feel ready for the next day's work.

Items were rated on a five-point Likert scale from “not at all” to “almost always.”

The work domain was represented by five categorical variables: *worktime* (full-time/part-time), *work position* (manager/subordinate), *employee responsibility* (supervisor/employee), *paid work time* (<35 h/36–45 h/>46 h), and *unpaid work time* (0 h/1–5 h/6–10 h/>10 h). The two variables, *work position* and *employee responsibility* were both used, since being a manager does not always include employee responsibilities. This is especially true for managers higher up in a corporate structure while first line managers are often those with employee responsibilities. According to Cerdas et al. (2019), *industry* is categorized into seven categories: education, health, and social care, labor-intensive services, knowledge-intensive services, public administration, goods and energy production, and machinery operations. Public administration is set as a reference since that industry was the most gender balanced (Cerdas et al., 2019).

Private life domain includes two categorical variables, i.e., *relationship status* (single or married/cohabiting) and *children* living at home (children/no children), and one continuous variable, namely *subjective social status* (SSS). SSS is represented by self-rated socioeconomic status for gainfully employed and it is measured using the MacArthur ladder scale (Miyakawa et al., 2012) comprising 10 graphical rungs. SSS was transformed into a categorical variable based on quartiles.

### Analytical Strategy

In line with the first part of the aim, a confirmatory factor analysis (CFA) was run to determine the factorial structure of work–family enrichment. Thereafter, a multigroup confirmatory factor analysis (MCFA) with measurement invariance was performed to assess the possible gender differences in the factorial structure of enrichment. Three indices of the goodness of fit were used to evaluate the model fit: the root mean square error of approximation (RMSEA), standardized root mean square residual (SRMR), and the comparative fit index (CFI). Typically, the CFI is used with a value >0.95 along with SRMR (good models <0.08) and RMSEA (good models <0.06). Chi-square statistics were used to confirm significant differences in the factorial structure between the structural models.

Next and in line with the second part of the aim, the level of enrichment and variable distribution across gender were explored using independent *t*-test and chi-square test. Lastly, a fixed effect linear mixed model (LMM) analysis was performed to explore the likelihood of experiencing enrichment by each of the variables in the work and private life domain. Since studies indicate that management roles of men and women differ across industries, mainly in terms of the number of employees, additional models were run to identify interaction effects between industry and responsibility over other workers (supervisor/employee) and work position (manager/subordinate). The LMM was carried out using restricted maximum likelihood (REML) model fit, which is presented by Schwarz's Bayesian information criterion (BIC) for men and women separately.

Factor analyses were run with Mplus version 8. All other analyses were executed using IBM SPSS 25.0.

## Results

### Gender as a Key Role in the Construct of Enrichment

First, a CFA was carried out for the whole sample (*n* = 11,553), and this confirmed the best model fit for a two-factor correlated model (x^2^ [8], 656.570; *p* < 0.001; RMSEA = 0.085; SRMR = 0.036; CFI = 0.978). The results confirm the previous findings of a two-factor structure representing the two directions of enrichment, namely WLE and LWE (Greenhaus and Beutell, 1985). Next, the MCFA was run to explore possible differences in the factorial structure between men and women. The results indicate metric invariance (x^2^ [24], 713.320; RMSEA = 0.071; SRMR = 0.038; CFI = 0.977), while a strong invariance is not given (*p* < 0.0001).

**Figure 1:**
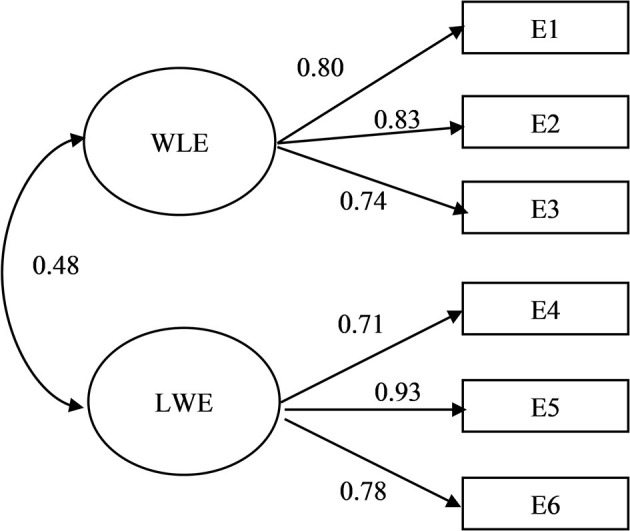
Factor loading/correlations for a two-factor model of enrichment for men.

**Figure 2:**
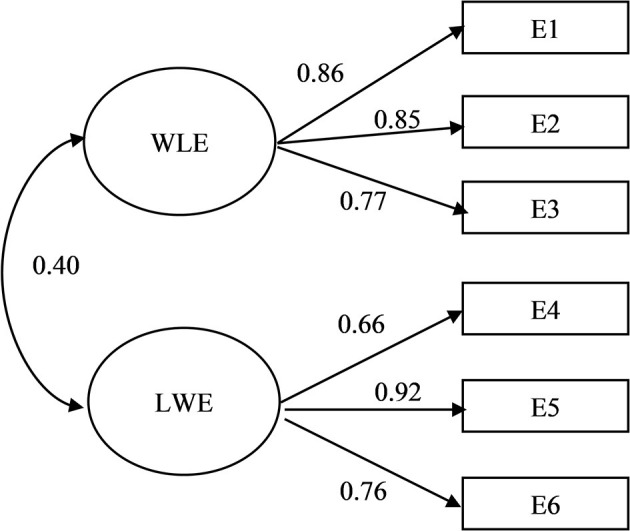
Factor loading/correlations for a two-factor model of enrichment for women.

Although the independent contribution of each of the variables representing WLE (E1, E2, and E3) was rather similar between genders, factor loadings were generally higher, though marginally, for women than for men (Figures 1, 2). A similar pattern was observed for LWE (E4, E5, and E6). Item E4 (I am in a better mood at work because of everything I have going for me in my personal life) loaded substantially higher for men than women (0.71 and 0.66, respectively). The relationship between the two factors of enrichment was higher for men (0.48) than women (0.40). A reliability test showed high internal consistency for the respective factors and respective gender: WLE (Cronbach's alpha = 0.83 for men and Cronbach's alpha = 0.85 for women) and LWE (Cronbach's alpha = 0.84 for men and Cronbach's alpha = 0.81 for women).

### The Relationship Between Enrichment and Factors in the Domains of Work and Private Life That Have a Strong Gender Cultural Norm

Depicting each variable by gender, a pattern of manifest-gendered structures in work and private life emerged (Supplementary Table 1). All variables, except WLE, showed significant differences between men and women. Men experienced a marginally (but not significantly) higher level of WLE (1.46 and 1.43), while women experienced higher levels of LWE (2.32 and 2.26). More men than women had a full-time contract and they more often reported working longer hours. Men more often found themselves in a management position and had supervisory responsibility over employees. Women reported spending more time on housework than men. Women were more often found in industries that are female-dominated, while the situation was reversed for men. Labor- and knowledge-intensive services were the most gender equal industries. Moving to the private domain, more men than women had children living at home and had a partner. Moreover, more men belonged in the high and middle socioeconomic status groups.

Focusing on WLE (Table 1), overall, BIC numbers presented significantly a better model fit for men than for women. Thus, the included variables better explained the level of WLE in men than in women. Men who reported working over 45 h a week also reported the highest level of WLE compared to those working 36 to 45 h. No significant differences were observed between women working long vs. shorter hours. Being in a management position as well as having a supervisory role increased the likelihood of reporting high WLE among men and women. Across different industries, only men and women working in goods and energy reported significantly lower levels of WLE than those in the public administration. Women who reported doing 0 h of housework report significantly lower levels of WLE. However, only 0.1% of the women reported 0 h of housework (see Supplementary Material), and those who did report 0 h might suffer health issues that diminish their possibility to do housework and might therefore experience lower levels of enrichment.

**Table 1:** Linear mixed model for men and women separately for the likelihood of reporting work-life enrichment (WLE).

	**Men**		**Women**	
	**Estimate**	**Std error**	**Estimate**	**Std error**
**Work domain**				
Full-time contract	−0.068	0.042	−0.012	0.025
Bayesian information criterion (BIC)		11,132		15,529
**Work hours**				
<35 h	0.056	0.042	0.021	0.039
36–45 h	−0.105^*^	0.031	−0.022	0.036
>45	Ref		Ref	
BIC		12,200		16,897
Manager	0.175^*^	0.030	0.185^*^	0.033
BIC		11,809		16,448
Supervisor	0.102^*^	0.027	0.128^*^	0.026
BIC		11,828		16,454
**Industry**				
Public administration	Ref		Ref	
Education	−0.011	0.065	−0.037	0.042
Health and social care	0.010	0.065	−0.024	0.039
Labor-intensive services	−0.002	0.054	0.016	0.044
Knowledge-intensive services	−0.014	0.053	0.069	0.048
Machinery operations	−0.018	0.053	0.062	0.068
Goods and energy production	−0.139^*^	0.051	−0.203^*^	0.054
BIC		12,236		17,053
**Housework hours**				
0 h	−0.031	0.090	−0.699^*^	0.337
1–5 h	−0.002	0.046	0.041	0.030
6–10 h	−0.002	0.047	0.026	0.028
11 h−15 h	Ref		Ref	
BIC		12,330		17,118
**Private-life domain**				
Children at home	−0.008	0.025	−0.002	0.022
BIC		12,221		17,030
Living with a partner	0.113^*^	0.032	0.007	0.026
BIC		12,309		17,157
**Subjective social status (SSS)**				
Low	−0.472^*^	0.030	−0.384^*^	0.027
Mid	−0.208^*^	0.030	−0.210^*^	0.028
High	Ref		Ref	
BIC		11,797		16,545

* *Sig > 0.05*.

For men, but not for women, living with a partner was associated with higher levels of WLE (Table 1). Both men and women belonging to the highest SSS group reported the highest levels of enrichment, and differences were great especially in comparison to low and to middle SSS groups.

Moving to LWE presented in Table 2, as with WLE, the BIC numbers for LWE were generally lower for men than for women, indicating a significantly better model fit for men (Table 2). Women working full time reported higher levels of LWE than women working part time, whereas no significant difference was seen among men. For men, working more than 45 h was related to significantly higher levels of LWE than if they work normal work hours. Men and women in management positions reported experiencing more LWE than their subordinates, and for persons with supervisory responsibility, LWE was also higher. Similarly, to WLE, men working in goods and energy production also reported significantly lower levels of LWE. For women, those working in education reported the highest LWE levels, although estimates reached only borderline significance (*p* = 0.056). Time spent on housework had no significant relationship with LWE.

**Table 2:** Linear mixed model for men and women separately for the likelihood of reporting life-work enrichment (LWE).

	**Men**		**Women**	
	**Estimate**	**Std error**	**Estimate**	**Std error**
**Work domain**				
Full-time contract	0.032	0.042	0.093^*^	0.024
BIC		11,160		14,946
**Work hours**				
<35 h	−0.067	0.043	−0.053	0.037
36–45 h	−0.061^*^	0.031	0.013	0.034
>45	Ref		Ref	
BIC		12,252		16,314
Manager	0.140^*^	0.031	0.140^*^	0.032
BIC		11,920		15,897
Supervisor	0.119^*^	0.026	0.119^*^	0.025
BIC		11,922		15,494
**Industry**				
Public administration	Ref		Ref	
Education	−0.047	0.065	0.076^a^	0.040
Health and social care	−0.029	0.066	0.010	0.038
Labor-intensive services	−0.025	0.054	−0.035	0.038
Knowledge-intensive services	−0.048	0.053	−0.007	0.042
Machinery operations	−0.094	0.053	−0.085	0.065
Goods and energy production	−0132^*^	0.052	−0.064	0.051
BIC		12,343		16,501
**Housework hours**				
0 h	−0.147	0.090	−0.530	0.322
1–5 h	−0.052	0.046	0.037	0.028
6–10 h	−0.002	0.048	0.051	0.027
11–15 h	Ref		Ref	
BIC		12,386		16,511
**Private-life domain**				
Children at home	0.063^*^	0.025	−0.027	0.021
BIC		12,290		16,433
Living with a partner	0.410^*^	0.031	0.230^*^	0.025
BIC		12,224		16,493
**Subjective social status (SSS)**				
Low	−0.339^*^	0.031	−0.362^*^	0.026
Mid	−0.119^*^	0.031	−0.161^*^	0.027
High	Ref		Ref	
BIC		11,965		16,025

* *Sig > 0.05;*

a *sig = 0.056*.

Men, but not women, with children, reported significantly higher levels of LWE than those without children (Table 2). Men and women living with a partner showed significantly and essentially higher levels of LWE than men and women who live alone. It could also be seen that for men, the difference in the level of LWE between living with a partner vs. living alone is larger than for women. Socioeconomic groups affected the level of LWE. Men and women belonging to the higher level of SSS groups also reported higher levels of LWE, and the differences between the groups were considerable.

In Table 3, interaction effects between industry and work position (manager/subordinate), and employee responsibilities (supervisor/employee), respectively, are presented. For LWE, female managers in labor-intensive services reported significant and much higher levels of enrichment compared to their subordinates. Turning to WLE, the results showed no significant relationships between WLE and either work position or supervisory responsibilities for men. For women, adding the effect of employee responsibility to industries showed that in health and social care as well as in knowledge-intensive services, women in management positions reported significant and considerably higher levels of enrichment than their subordinates.

**Table 3:** The interaction between industry and work position, respectively, and employee responsibility for men and women in work-life enrichment (WLE) and life-work enrichment (LWE).

	**WLE**				**LWE**			
	**Men**		**Women**		**Men**		**Women**	
	**Est**.	**Std E**	**Est**.	**Std E**	**Est**.	**Std E**	**Est**.	**Std E**
**Work position (Management position)**								
Public administration	Ref		Ref		Ref		Ref	
Education	−0.145	0.176	0.159	0.128	0.097	0.180	0.085	0.123
Health and social care	−0.046	0.180	0.192	0.114	0.105	0.183	0.121	0.110
Labor-intensive services	−0.090	0.129	0.149	0.114	−0.016	0.131	0.245^*^	0.109
Knowledge-intensive services	−0.083	0.127	0.011	0.124	−0.006	0.129	−0.060	0.0119
Machinery operations	−0.082	0.129	0.094	0.187	0.054	0.131	0.236	0.179
Goods and energy production	−0.213	0.125	−0.087	0.147	−0.061	0.127	0.020	0.141
BIC	11,716		16,300		11,878		15,766	
**Employee responsibilities (Supervisory responsibilities)**								
Public administration	Ref		Ref		Ref		Ref	
Education	−0.145	0.145	0.150	0.097	0.070	0.148	−0.001	0.094
Health and social care	−0.132	0.139	0.271^*^	0.089	0.114	0.142	0.107	0.086
Labor-intensive services	−0.125	0.112	0.193^*^	0.098	−0.027	0.0114	0.084	0.094
Knowledge-intensive services	−0.110	0.108	0.125	0.105	−0.033	0.110	−0.052	0.101
Machinery operations	−0.080	0.110	0.230	0.159	0.018	0.112	0.071	0.153
Goods and energy production	−0.184	0.106	0.146	0.245	−0.028	0.108	−0.026	0.121
BIC	11,732		16,302		11,877		15,769	

* *Sig > 0.05*.

## Discussion

By taking a gender-theoretical approach, this study aimed to explore the role of gender in enrichment. Initially, a two-factor structure of enrichment was confirmed for both men and women, representing the two directions of enrichment, WLE, and LWE. Our confirmation of the two-factorial structures for men and women corresponds to the existing research and definitions (Carlson et al., 2006; Greenhaus and Powell, 2006). However, what this study adds to the previous knowledge of the two-factorial structures of enrichment is the clear pattern of social constructs of gender and gendered cultural expectations, a pattern that we would not have looked for if we had not been guided by gender theory. Our results show that men and women do not interpret or value items measuring enrichment in the same way. Therefore, because of the way enrichment is measured at present, it is questionable whether one can compare the level of enrichment of men and women in Sweden using the scale of Fisher et al. (2009). When linking factors representing the work domain and the private life domain with WLE and LWE, respectively, continuing strong evidence for acknowledging the cultural construct of gender in enrichment and factors associated with enrichment is shown. In the meta-analysis conducted by Lapierre et al. (2018), it was concluded that the relationship between enrichment and work and family factors was not moderated by gender. However, by including gender as a moderator, it was assumed that men and women construct enrichment in a similar matter, which we have shown is not the case. Men and women must be viewed in their social context and in relation to the construction of gender (Connell, 2002). In the doing of gender, men and women reproduce gender by exercising tasks with respective masculine and feminine cultural norms (West and Zimmerman, 1987). This implies different meanings in tasks for men and women, and also shows that the capabilities of men and women to exercise tasks, such as management are viewed differently. Gender cultural norms in work and private life seem to have implications for the two dimensions related to enrichment. Thus, the gendered cultural norms in work and private life manifest in the experience of enrichment as well as in the construct of enrichment. Our results extend previous theories of enrichment (Greenhaus and Powell, 2006) by presenting how enrichment is embodied—that is, how the social construct of genders, the historical contexts, and the material structures are incorporated in how men and women value and experiences enrichment. Further development is needed for the instruments of enrichment where the manifestation of gender cultural norms and the organization of genders in both the work domain and the private life domain are considered.

This study was carried out in a setting that is often considered to be gender equal (Thévenon, 2011). However, in Sweden, the private life domain still has strong feminine cultural norms, and narrations of equality are mainly directed toward the labor market (Hagqvist, 2016; Carbin et al., 2017). Women often find themselves balancing between being the main career and being equal (in terms of participation in the work life), which seems to have a negative effect on their well-being (Hagqvist, 2016). In a qualitative study of Scandinavian managers, the authors found that female interviewees strongly identified with the masculine norm as the good worker while they also emphasized the importance of support from family and friends to uphold this role as a good worker (Hagqvist et al., 2020). To find broader support for the phenomenon of the embodiment of enrichment, this study should be replicated in other countries and settings.

Focusing on the various factors related to enrichment that are highlighted in this study, four factors stand out. The first two concern the work domain. First, as expected and following the masculine norm, men working more than 45 h a week reported higher enrichment (both WLE and LWE) than those working approximately full time (40 h a week in Sweden). This suggests that men in this situation strengthen their masculine capital, the advantages that follow from the social construction of femininities and masculinities (Huppatz, 2009) in work, which seems to contribute to the experience of enrichment. Secondly, women with a full-time contract experience higher levels of LWE than those with part-time contract. The data from the study show that Swedish women more often have part-time contracts than men (Statistics Sweden, 2020). A part-time contract often implies less income and more domestic work and care of children, which are factors that strengthen feminine capital (Huppatz, 2009). Thus, as in the study of the Scandinavian managers (Hagqvist et al., 2020), women seem to benefit from adhering to masculine capital. The two last factors concern the domestic domain, having a partner and children at home. In general, having a partner seems more important for the level of enrichment for men than that for women. While both men and women living with a partner report significantly more LWE than those living alone, only the relationship status of men was important for WLE. Men with a partner report higher WLE. These results partly confirm and give nuance to previous findings by Lapierre et al. (2018). The fact that a partner is more important for the level of enrichment for men indicates that they seem to find more support and satisfaction from their relationship, which in turn enriches their work. As satisfaction in partnership can be related to the division of housework (Harryson et al., 2016), it would be interesting to further study the relationship between enrichment and perceived fairness, division of housework, and gender equal attitudes. Moreover, men and not women, present a relationship between having a partner and WLE. These results can stem from the notion that Swedish men still feel satisfaction in supplying their family with an income while women still struggle to manage the work domain and family domain (Hagqvist, 2016). Although having children living at home is an important factor influencing the level of interference (Gallie and Russell, 2009), it has little importance for the level of enrichment. A significant difference was only observed in the level of LWE for men. Although the effect was small, it is interesting as it is often argued that women benefit more from domestic factors than men (Lapierre et al., 2018). Perhaps since women are more involved in childcare (Hagqvist et al., 2020), they benefit less from children in their working life. This should be further explored in future research. In the division of work, there is constant negotiation within the family concerning who will do what. In this negotiation, a gender power relation has implications for the narrations constructing the roles of male and female in the family.

Another important result is the relationship between enrichment and self-reported SSS. Self-reported SSS seems to stand out as the single factor that most strongly relates to enrichment. Although SSS is positively related to enrichment for both men and women, the results indicate that for men, to a higher degree than for women, the likelihood of reporting high WLE differs quite substantially between the high and the low SSS group. Those who report belonging to the highest SSS group report substantially higher levels of enrichment, both in terms of WLE and LWE. Income and status are more closely linked to masculinities than femininities (Ely and Meyerson, 2000; Connell, 2008) and could therefore have greater implications for the relationship between socioeconomic status and enrichment for men than for women. In general, our regression model showed a better model fit for men than for women, which indicates that the included variables to a greater extent explain the enrichment level of men than that of the women. Further studies are required to find what best explains the level of enrichment for women. Although not conclusive, the results indicate some degree of domain-specific interplay with enrichment. In the work domain, having a full- or part-time contract, industry, managerial positions, and the number of hours worked are areas where the powerful relationship between men and women becomes visualized (Connell, 2006; Hagqvist, 2016).

It has been empirically substantiated that managerial positions imply different preconditions and roles for men and women across different industries (Björk and Härenstam, 2016). Although little difference was observed across industries, some differences were observed in the interaction terms. Men working in goods and energy production report significantly lower levels of WLE and LWE than men in other industries. Women working in goods and energy production report significantly lower WLE than other women, while women in education reported higher LWE. In the moderating effect of industry and management and employee responsibility, patterns of gendered cultural norms become visualized. Female managers in female dominated industries tend to have higher levels of enrichment. These results stand in contrast to those of Björk and Härenstam (2016) who showed that first line managers in female- dominated industries often felt higher demands and lower support from the organization. Furthermore, studies show that women in professional jobs and those with long work hours more often report conflicting demands between work and private life (McGinnity and Calvert, 2009; Bianchi and Milkie, 2010). Meanwhile, our results align with the findings by Hagqvist et al. (2020) who showed that women managers could strengthen their self-identity and experience more enrichment by adhering to the masculine norms and culture in management. However, they also found that these women experienced hardship in relation to the family (Hagqvist et al., 2020). Thus, it seems to be a struggle and hardship for women in leading positions. However, whether this hardship overrules the role of salience for women in management positions and those women with supervisory responsibilities is yet to be further studied. Furthermore, future studies should focus on the balance and imbalance between work and private life experienced by these women and how organizations can support women to strive without hardship.

### Strengths and Limitations

Gender is a complex continuum of femininities and masculinities and should not be marginalized to the dichotomy of men and women. Although in statistics this continuum is difficult to measure, we have tried to find variances both within and across genders. Although the intersectional power structures existing among gender, class, and ethnicity are strongly postulated in literature (Ely and Meyerson, 2000; Acker, 2006), we have not taken ethnicity into consideration. This is because the number of respondents with a non-Swedish background is fairly small in the sample. The role of intersectionality in enrichment should therefore be further acknowledged in future studies.

## Conclusion

This study also expands on the theories of enrichment to include how the social construct of gender, the historical contexts, and the material and structures are incorporated in the experience of enrichment by men and women and the value they attach to it; that is, how gender is embodied in enrichment and the factors related to enrichment. The gendered aspects of work and private life should be considered in future enrichment studies.

## Data Availability Statement

Given the restrictions we have from the ethical review board and considering that sensitive personal data protected by GDPR and by secrecy following from the Swedish Public Access to Information and Secrecy Act are handled, we are not able to make the data freely available. The data may only be provided to other researchers in line with Swedish law and after consultation with the Stockholm University legal department. Researchers wishing to access the minimal de-identified data set should make a formal request to Stockholm University at https://constanze.leineweber@su.se.

## Author Contributions

EH has contributed with conception and design of the study and drafting the article. EH and CL have in joint collaboration conducted the data analysis and interpretation. CL and AN have made critical revisions of the article and approved its submission. All authors contributed to the article and approved the submitted version.

## Conflict of Interest

The authors declare that the research was conducted in the absence of any commercial or financial relationships that could be construed as a potential conflict of interest.

## References

[B1] Acker J. (1990). Hierarchies, jobs, bodies: a theory of gendered organizations. Gender Soc. 4, 139–158. 10.1177/089124390004002002

[B2] Acker J. (1992). From sex roles to gendered institutions. Contemp. Sociol. 21, 565–569. 10.2307/2075528

[B3] Acker J. (2006). Inequality regimes gender, class, and race in organizations. Gender Soc. 20, 441–464. 10.1177/0891243206289499

[B4] Albrecht S. C. Kecklund G. Tucker P. Leineweber C. (2016). Investigating the factorial structure and availability of work time control in a representative sample of the Swedish working population. Scand. J. Public Health 44, 320–328. 10.1177/1403494815618854 26620363 PMC4819796

[B5] Allen T. D. Martin A. (2017). The work-family interface: a retrospective look at 20 years of research in JOHP. J. Occup. Health Psychol. 22, 259–272. 10.1037/ocp0000065 28150990

[B6] Arendell T. (2000). Conceiving and investigating motherhood: the decade's scholarship. J. Marriage Fam. 62, 1192–1207. 10.1111/j.1741-3737.2000.01192.x

[B7] Beham B. Drobni,č S. Präg P. Baierl A. Lewis S. (2020). Work-to-family enrichment and gender inequalities in eight European countries. Int. J. Hum. Resour. Manage. 31, 589–610. 10.1080/09585192.2017.1355837

[B8] Bernhardt E. Noack T. Lyngstad T. H. (2008). Shared housework in Norway and Sweden: advancing the gender revolution. J. Eur. Soc. Policy 18, 275–288. 10.1177/0958928708091060

[B9] Bianchi S. M. Milkie M. A. (2010). Work and family research in the first decade of the 21st century. J. Marr. Fam. 72, 705–725. 10.1111/j.1741-3737.2010.00726.x31294385

[B10] Björk L. Härenstam A. (2016). Differences in organizational preconditions for managers in genderized municipal services. Scand. J. Manage. 32, 209–219. 10.1016/j.scaman.2016.09.002

[B11] Cantalini S. Härkönen J. Dahlberg J. (2017). Does Postponing Pay Off? Timing of Parenthood, Earnings Trajectories, and Earnings Accumulation in Sweden 1990-2012. Stockholm University, Stockholm Research Reports in Demography.

[B12] Carbin M. Overud J. Kvist E. (2017). Feminism Somlönearbete [Feminins as Paid Work]. Stockholm: Leopard förlag.

[B13] Carlson D. S. Kacmar K. M. Wayne J. H. Grzywacz J. G. (2006). Measuring the positive side of the work-family interface: development and validation of a work-family enrichment scale. J. Vocat. Behav. 68, 131–164. 10.1016/j.jvb.2005.02.002

[B14] Cerdas S. Härenstam A. Johansson G. Nyberg A. (2019). Development of job demands, decision authority and social support in industries with different gender composition–Sweden. 1991–2013. BMC Public Health 19:758. 10.1186/s12889-019-6917-8 31200675 PMC6570932

[B15] Cinamon R. G. Rich Y. (2002). Gender differences in the importance of work and family roles: implications for work–family conflict. Sex Roles 47, 531–541. 10.1023/A:1022021804846

[B16] Connell R. (2002). Gender. Cambridge: Polity.

[B17] Connell R. (2006). Glass ceilings or gendered institutions? Mapping the gender regimes of public sector worksites. Public Administr. Rev. 66, 837–849. 10.1111/j.1540-6210.2006.00652.x

[B18] Connell R. (2008). Maskuliniteter (Å. Lindén, Trans.). Göteborg: Daidalos.

[B19] Connell R. Messerschmidt J. W. (2005). Hegemonic masculinity rethinking the concept. Gender Soc. 19, 829–859. 10.1177/0891243205278639

[B20] DeVault M. L. (1991). Feeding the Family: The Social Organization of Caring as Gendered Work. Chicago: University of Chicago Press.

[B21] Dribe M. Stanfors M. (2009). Does parenthood strengthen a traditional household division of labor? Evidence from Sweden. J. Marr. Fam. 71, 33–45. 10.1111/j.1741-3737.2008.00578.x

[B22] Eek F. Axmon A. (2014). Gender inequality at home is associated with poorer health for women. Scand. J. Public Health 43, 176–182. 10.1177/1403494814562598 25504654

[B23] Ellingsæter A. L. (2014). Skandinavia: de mest kjønnsdeltearbeidsmarkedene? [Scandinavia: the most gender segregated labour markets?], in Jämställtarbete? Organisatoriskaramarochvillkoriarbetslivet. [Equal Work? Organizational Frames and Conditions in Work Life]. Swedish Government Official Reports, SOU 2014:30, 23–44.

[B24] Elvin-Nowak Y. Thomsson H. (2001). Motherhood as idea and practice a discursive understanding of employed mothers in Sweden. Gender Soc. 15, 407–428. 10.1177/089124301015003005

[B25] Ely R. J. Meyerson D. E. (2000). Theories of gender in organizations: A new approach to organizational analysis and change. Res. Organ. Behav. 22, 103–151. 10.1016/S0191-3085(00)22004-2

[B26] Fahlén S. (2014). Does gender matter? Policies, norms and the gender gap in work-to-home and home-to-work conflict across Europe. Commun. Work Fam. 17, 371–391. 10.1080/13668803.2014.899486

[B27] Fisher G. G. Bulger C. A. Smith C. S. (2009). Beyond work and family: a measure of work/nonwork interference and enhancement. J. Occup. Health Psychol. 14:441. 10.1037/a0016737 19839663

[B28] Fogelberg Eriksson A. (2016). Chefers föreställningar om ledarskap och kön, in Mot ett Förändrat Ledarskap? Om Chefers Arbete och Ledarskap i ett Organisationsperspektiv, eds P.-E. Ellström, A. Fogelberg Eriksson, H. Kock, and A. Wallo (Lund: Studentlitteratur), 97–118.

[B29] Gallie D. Russell H. (2009). Work-family conflict and working conditions in Western Europe. Soc. Indic. Res. 93, 445–467. 10.1007/s11205-008-9435-0

[B30] Greenhaus J. H. Beutell N. J. (1985). Sources of conflict between work and family roles. Acad. Manage. Rev. 10, 76–88. 10.5465/amr.1985.4277352

[B31] Greenhaus J. H. Powell G. N. (2006). When work and family are allies: a theory of work-family enrichment. Acad. Manage. Rev. 31, 72–92. 10.5465/amr.2006.19379625

[B32] Haas L. Allard K. Hwang P. (2002). The impact of organizational culture on men's use of parental leave in Sweden. Commun. Work Fam. 5, 319–342. 10.1080/1366880022000041801

[B33] Hagqvist E. (2016). The juggle and struggle of everyday life. Gender, division of work, work-family perceptions and well-being in different policy contexts [Ph.D.thesis]. Mid Sweden University, Östersund, Sweden.

[B34] Hagqvist E. GillanderGådin K. Nordenmark M. (2012). Division of labor, perceived labor-related stress and well-being among European couples. Open J. Prev. Med. 2, 452–460. 10.4236/ojpm.2012.24064

[B35] Hagqvist E. Nordenmark M. Pérez G. Trujillo Alemán S. GillanderGådin K. (2017). Parental leave policies and time use for mothers and fathers: a case study of Spain and Sweden. Soc. Health Vulnerabil. 8:1374103. 10.1080/20021518.2017.1374103

[B36] Hagqvist E. Toivanen S. Vinberg S. (2019). The gender time gap: time use among self-employed women and men compared to paid employees in Sweden. Time Soc. 28, 680–696. 10.1177/0961463X16683969

[B37] Hagqvist E. Vinberg S. Tritter J. Q. Wall E. Landstad B. J. (2020). The same, only different: doing management in the intersection between work and private life for men and women in small-scale enterprises. Work Employ. Soc. 34, 262–280. 10.1177/0950017019871244

[B38] Härenstam A. (2009). Exploring gender, work and living conditions, and health–suggestions for contextual and comprehensive approaches. Scand. J. Work Environ. Health 35, 127–133. 10.5271/sjweh.1308 19294318

[B39] Harryson L. Aléx L. Hammarström A. (2016). I have surly passed a limit, it is simply too much: women's and men's experiences of stress and wellbeing when living within a process of housework resignation. BMC Public Health 16:224. 10.1186/s12889-016-2920-5 26944701 PMC4779260

[B40] Harryson L. Novo M. Hammarström A. (2012). Is gender inequality in the domestic sphere associated with psychological distress among women and men? Results from the Northern Swedish Cohort. J. Epidemiol. Commun. Health 66, 271–276. 10.1136/jech.2010.109231 20940171

[B41] Hirdman Y. (1990). Genussystemet, in Demokrati och makt i Sverige: Maktutredningen, SOU 1990:44.

[B42] Huppatz K. (2009). Reworking bourdieu's Capital': feminine and female capitals in the field of paid caring work. Sociology 43, 45–66. 10.1177/0038038508099097

[B43] Kling J. HolmqvistGattario K. Frisen A. (2017). Swedish women's perceptions of and conformity to feminine norms. Scand. J. Psychol. 58, 238–248. 10.1111/sjop.12361 28436998

[B44] Kvande E. Rasmussen B. (1994). Men in male-dominated organizations and their encounter with women intruders. Scand. J. Manage. 10, 163–173. 10.1016/0956-5221(94)90018-3

[B45] Lapierre L. M. Li Y. Kwan H. K. Greenhaus J. H. DiRenzo M. S. Shao P. (2018). A meta-analysis of the antecedents of work-family enrichment. J. Organ. Behav. 39, 385–401. 10.1002/job.2234

[B46] Magnusson Hanson L. I Leineweber C. Persson V. Hyde M. Theorell T.. (2018). Cohort profile - the Swedish Longitudinal Occupational Survey of Health (SLOSH). Int. J. Epidemiol. 47, 691–692. 10.1093/med/9780190238308.003.0004 29340637 PMC6005080

[B47] Martin J. (2011). Does gender inequality ever disappear? in Handbook of Gender, Work and Organization, eds E. L. Jeanes, D. Knights, and P. Yancey Martin (Chichester: Wiley), 213–230.

[B48] McGinnity F. Calvert E. (2009). Work-life conflict and social inequality in Western Europe. Soc. Indic. Res. 93, 489–508. 10.1007/s11205-008-9433-2

[B49] Miyakawa M. Magnusson Hanson L. L. Theorell T. Westerlund H. (2012). Subjective social status: its determinants and association with health in the Swedish working population (the SLOSH study). Eur. J. Public Health 22, 593–597. 10.1093/eurpub/ckr064 21646364

[B50] Nyberg A. Leineweber C. Hanson L. M. (2015). Gender differences in psychosocial work factors, work–personal life interface, and well-being among Swedish managers and non-managers. J. Int. Arch. Occup. Environ. Health 88, 1149–1164. 10.1007/s00420-015-1043-0 25761632

[B51] Ruppanner L. Branden M. Turunen J. (2018). Does unequal housework lead to divorce? Evidence from Sweden. Sociology 52, 75–94. 10.1177/0038038516674664

[B52] Staland-Nyman C. Alexanderson K. Hensing G. (2008). Associations between strain in domestic work and self-rated health: a study of employed women in Sweden. Scand. J. Public Health 36, 21–27. 10.1177/1403494807085307 18426781

[B53] Statistics Sweden (2020). Women and Men in Sweden 2020 Facts and Figures. Stockholm: Statistics Sweden.

[B54] Thévenon O. (2011). Family policies in OECD countries: a comparative analysis. Popul. Dev. Rev. 37, 57–87. 10.1111/j.1728-4457.2011.00390.x 21714199

[B55] West C. Fenstermaker S. (1995). Doing difference. Gender Soc. 9, 8–37. 10.1177/089124395009001002

[B56] West C. Zimmerman D. H. (1987). Doing gender. Gender Soc. 1, 125–151. 10.1177/0891243287001002002

[B57] Williams J. C. Berdahl J. L. Vandello J. A. (2016). Beyond work-life integration. Annu. Rev. Psychol. 67, 515–539. 10.1146/annurev-psych-122414-033710 26442669

