# Supplementary material for: A Theoretical Development of the Gender Embodiment of Enrichment: A Study of Gender Norms in Enrichment and Factors Related to Enrichment in a Sample of the Swedish Working Population
Source: Front Sociol. 2021 Apr 29;6:669789. doi: 10.3389/fsoc.2021.669789 (PMC8116733; doi:10.3389/fsoc.2021.669789)
Supplement: Supplementary file 1 [file Table_1.docx]

Supplementary material

Table 1. Mean of work-life enrichment (WLE) respectively life-work enrichment (LWE) for men and women as well as distributions (%) and significant differences by gender across each variable in the work and private-life domains.

|  | Within genders | | Between genders | |  |
| --- | --- | --- | --- | --- | --- |
|  | Men | Women | Men | Women | Sig. Dif. |
| WLE (mean) | 1.46 | 1.43 |  |  |  |
| LWE (mean) | 2.26 | 2.32 |  |  | * |
| **Work domain** |  |  |  |  |  |
| Full-time | 89.1 | 68.6 | 48.6 | 51.4 | * |
| Part-time | 10.9 | 31.4 | 20.2 | 79.8 | * |
| Paid work hours: |  |  |  |  | * |
| > 35h | 14.3 | 29.6 | 26.3 | 73.7 |  |
| 36-45 | 63.4 | 59.1 | 44.1 | 55.1 |  |
| < 45 | 22.3 | 11.3 | 59.2 | 40.8 |  |
| Manager | 22.4 | 12.7 | 56.3 | 43.7 | * |
| Subordinate | 77.6 | 87.3 | 39.4 | 60.6 | * |
| Supervisor | 34.7 | 24.9 | 50.5 | 49.5 | * |
| Employee | 65.3 | 75.1 | 38.9 | 61.1 | * |
| Industry: |  |  |  |  | * |
| Public administration | 8.0 | 10.3 | 36.3 | 63.7 |  |
| Education | 7.0 | 21.1 | 19.5 | 80.5 |  |
| Health and social care | 6.8 | 31.1 | 13.7 | 86.3 |  |
| Labor intensive services | 16.6 | 16.4 | 42.4 | 57.6 |  |
| Knowledge intensive services | 19.0 | 10.6 | 56.6 | 43.4 |  |
| Machinery operations | 19.2 | 3.4 | 80.3 | 19.7 |  |
| Goods and energy production | 23.5 | 7.1 | 70.9 | 29.1 |  |
| Unpaid work hours: |  |  |  |  | * |
| 0h | 2.6 | 0.1 | 94.7 | 5.3 |  |
| 1-5h | 53.5 | 32.1 | 55.0 | 45.0 |  |
| 6-10h | 35.1 | 43.3 | 37.3 | 62.7 |  |
| 11-15h | 8.8 | 24.5 | 20.8 | 79.2 |  |
| **Private-life domain** |  |  |  |  |  |
| Children living at home | 55.1 | 43.0 | 43.3 | 56.7 | * |
| No children living at home | 44.9 | 57.0 | 41.4 | 58.6 | * |
| Living with partner | 80.9 | 77.6 | 43.3 | 56.7 | * |
| Living alone | 19.1 | 22.4 | 38.4 | 61.6 | * |
| Subjective social status (SSS): |  |  |  |  | * |
| Low | 34.5 | 39.5 | 39.0 | 61.0 |  |
| Mid | 32.1 | 33.2 | 41.4 | 58.6 |  |
| High | 33.3 | 27.2 | 47.3 | 52.7 |  |
